# Characterization of a Very Short Meq Protein Isoform in a Marek’s Disease Virus Strain in Japan

**DOI:** 10.3390/vetsci11010043

**Published:** 2024-01-20

**Authors:** Yoshinosuke Motai, Shiro Murata, Jumpei Sato, Akihito Nishi, Naoya Maekawa, Tomohiro Okagawa, Satoru Konnai, Kazuhiko Ohashi

**Affiliations:** 1 Laboratory of Infectious Diseases, Department of Disease Control, Faculty of Veterinary Medicine, Hokkaido University, Kita-18, Nishi-9, Kita-ku, Sapporo 060-0818, Japan; 2 Department of Advanced Pharmaceutics, Faculty of Veterinary Medicine, Hokkaido University, Kita-18, Nishi-9, Kita-ku, Sapporo 060-0818, Japan; 3 Chuo Livestock Hygiene Service Center, Agriculture Promotion Department, Kochi Prefecture, 3229 Otsu, Takaoka-cho, Tosa 781-1102, Japan; 4 Institute for Vaccine Research and Development (HU-IVReD), Hokkaido University, Kita-18, Nishi-9, Kita-ku, Sapporo 060-0818, Japan; 5 International Affairs Office, Faculty of Veterinary Medicine, Hokkaido University, Kita-18, Nishi-9, Kita-ku, Sapporo 060-0818, Japan

**Keywords:** Marek’s disease, Marek’s disease virus, *meq* gene, S-Meq, VS-Meq, deletion, transactivation, transrepression, Japanese strain

## Abstract

**Simple Summary:**

This study focuses on Marek’s disease virus (MDV), which causes malignant T-cell lymphomas and neurological disorders in chickens. The *meq* gene, an oncogene of MDV, plays an essential role in transformation through regulating the expression of host and viral genes. Previously, we reported that the deletion of the short isoform of Meq (S-Meq) decreased the pathogenicity of MDV. In this study, we identified a very short isoform of Meq (VS-Meq) in Japanese MDV strains and analyzed the effects of VS-Meq on transcriptional regulation using a reporter assay. The VS-Meq showed a 64-amino-acid (aa) deletion at the C-terminus. The reporter assays revealed that wild-type VS-Meq had a lower transrepression on the promoter of *pp38*, which is a viral antigen expressed in the cytolytic infection, whereas it did not affect the transactivation activities of the promoters of *meq* and *bcl-2*, an anti-apoptotic molecule. On the other hand, the 64-aa deletion did not affect the activity of the *pp38* promoter but enhanced the transactivation activities of the *meq* and *bcl-2* promoters. These findings suggest that the 64-aa deletion is involved in the functions of VS-Meq and highlight the need for further research on its effect on MDV pathogenicity.

**Abstract:**

Marek’s disease virus (MDV) causes malignant lymphoma (Marek’s disease; MD) in chickens. The Meq protein is essential for tumorigenesis since it regulates the expression of host and viral genes. Previously, we reported that the deletion of the short isoform of Meq (S-Meq) decreases the pathogenicity of MDV. Recently, we identified a further short isoform of Meq (very short isoform of Meq, VS-Meq) in chickens with MD in Japan. A 64-amino-acid deletion was confirmed at the C-terminus of VS-Meq. We measured the transcriptional regulation by VS-Meq in three gene promoters to investigate the effect of VS-Meq on protein function. Wild-type VS-Meq decreased the transrepression of the pp38 promoter but did not alter the transactivation activity of the Meq and Bcl-2 promoters. The deletion in VS-Meq did not affect the activity of the pp38 promoter but enhanced the transactivation activities of the Meq and Bcl-2 promoters. Collectively, the deletion of VS-Meq potentially enhanced the activity of the Meq promoter, while other amino acid sequences in wild-type VS-Meq seemed to affect the weak transrepression of the pp38 promoter. Further investigation is required to clarify the effects of these changes on pathogenicity.

## 1. Introduction

*Gallid alphaherpesvirus 2*, previously known as Marek’s disease virus (MDV) serotype 1, belongs to the genus *Mardivirus* and causes malignant T-cell lymphomas and neurological disorders, such as torticollis and ataxia in infected chickens (Marek’s disease, MD) [1]. The genome structure of MDV consists of two unique regions, unique long (UL) and unique short (US), and two duplicated repeat regions, internal/terminal repeat long (IRL/TRL) and internal/terminal repeat short (IRS/TRS), similar to those of other alphaherpesviruses such as herpes simplex virus and varicella zoster virus [2]. Cell-free MDV is produced in feather follicle epithelial cells, and chickens are infected with MDV through the respiratory tract [3]. During the acute phase of infection, MDV induces lytic infection in B and T lymphocytes and subsequently establishes latent infection in mainly infected CD4^+^ T cells. Finally, MDV transforms some of the latently infected cells and develops malignant lymphomas [1,2,4].

The occurrence of MD has been well-regulated by the introduction of live vaccines since the 1970s. A non-pathogenic strain of turkey herpesvirus (HVT, *Meleagrid alphaherpesvirus 1*, previously known as MDV serotype 3) was initially introduced as a vaccine against MD; subsequently, strains of *Gallid alphaherpesvirus 3* (previously called MDV serotype 2 and attenuated MDV serotype 1) were applied as vaccines to control MD [5]. Although these vaccines can prevent tumor formation, they cannot prevent infection or viral shedding. Their leaky vaccine properties cause the transmission and maintenance of highly virulent MDVs [6]. In addition, positive selection of these vaccines is thought to increase the genetic diversity of MDV field strains and cause the emergence of highly virulent MDVs [7]. Currently, the virulence of MDV strains is classified as mild (m)-to-virulent (v), very virulent (vv), and very virulent plus (vv+) MDV [1].

The *meq* gene is an oncogene of MDV present in the IRL and TRL regions and is one of the most highly mutated genes among MDV strains [8]. The *meq* gene product, Meq, plays a central role in MDV transformation, and *meq*-deleted MDV does not develop malignant lymphomas in infected chickens [9]. Meq serves as a transcription factor that resembles c-Jun (a member of the AP-1 family) and is involved in tumorigenesis by regulating the expression of host and viral genes [10,11]. Meq consists of a proline-/glutamine-rich region (Pro/Gln), basic region (BR), leucine zipper (ZIP) at the N-terminus, and transactivation domain at the C-terminus [12]. It can form a homodimer with Meq itself and heterodimers with basic leucine zipper (bZIP) proteins such as c-Jun, JunB, and c-Fos through the leucine zipper; dimerization is necessary for tumorigenesis [13]. The heterodimer with c-Jun can regulate the expression of several genes, such as *JTAP-1*, *HB-EGF*, *JAC*, *STAT-3*, and *bcl-2*, which are upregulated in Meq-transformed cells and are considered to contribute to transformation by MDV [11,14]. Chromatin immunoprecipitation sequencing analysis reveals that the Meq homodimer represses the expression of target genes by binding to a motif sequence (Meq responsible element II, MERE-II), whereas the Meq/c-Jun heterodimer activates the expression of genes associated with transformation by binding to AP-1 motif sequences [14]. The transactivation domain includes proline-rich regions and determines the level of transcriptional regulation. Differences in the amino acid sequences of Meq (such as polymorphisms, insertions, and deletions) affect transcriptional regulation and the pathogenicity of MDV [15,16,17]. Four consecutive prolines (PPPP) present in the Meq region of classical MDV strains are characteristic of this proline-rich region [18]. However, it remains unclear how PPPP sequences are associated with MDV pathogenicity. Most highly virulent MDV strains have distinct polymorphisms that disrupt the PPPP sequences, wherein the second proline residue in the PPPP sequence is substituted with other amino acids (PPPP to PXPP) [19]. Furthermore, insertions were detected in the transactivation domain of Meq in some MDV strains. CVI988/Rispens is the most common attenuated vaccine strain; it has a long isoform of Meq (L-Meq) with a 59/60-amino acid (aa) insertion in proline-rich regions, resulting in eight PPPP sequences [18]. However, a similar insertion was found in the proline-rich region of Meq of virulent MDV strains circulating in Australia, and PXPP substitutions were observed in the L-Meq proteins of these Australian strains [20]. Recent studies have shown that insertion into the proline-rich region increases the virulence of MDV [16,17], suggesting that the insertion contributes to the high virulence of MDV, whereas other factors, including polymorphisms in L-Meq, are involved in the low virulence of CVI988 [16,17,21].

In addition to the polymorphisms in Meq and insertions into proline-rich regions, we previously reported a short isoform of Meq (S-Meq) that includes a deletion in the proline-rich region, resulting in a decreased number of PPPP sequences [22]. Interestingly, the deletion decreases the virulence of MDV, although S-Meq shows a higher transactivation activity on the Meq promoter than on Meq [17]. These results indicate the contribution of different functions of proline-rich regions, ranging from transcriptional regulation to MDV pathogenicity. Recently, MDVs encoding S-Meq were reported in field isolates from Italy, Iraq, Iran, Saudi Arabia, and Japan [22,23,24,25]. Most MDVs with S-Meq were isolated from unvaccinated chickens or turkeys, and S-Meq contains a 41-amino-acid (aa) deletion in the proline-rich region. In 2022, a very short isoform of Meq (VS-Meq) was reported for the first time in unvaccinated backyard chickens in Iran as a field isolate [26]. The VS-Meq of the Iranian MDV strains contained one PPPP sequence, whereas the S-Meq contained two or three PPPP sequences. It is unknown if MDVs with VS-Meq are prevalent in other countries.

This study detected MDVs with VS-Meq in Japan. The VS-Meq detected in the Japanese MDV strains contained one PPPP sequence; however, the size of the deleted sequence was different from that of the Iranian MDV strains. To investigate whether the deletion found in the VS-Meq affected protein function, the effect of the deletion on transcriptional regulation by Meq was analyzed using a reporter assay.

## 2. Materials and Methods

### 2.1. Background of the Specimen

Unvaccinated chickens developed leg paralysis, respiratory symptoms, and diarrhea and died in Japan. Necropsy was performed at the Chuo Livestock Hygiene Service Center in Kochi Prefecture. Tumor-like masses were observed in the thoracic caves, and the spleens, sciatic nerves, and thoracic masses were collected. The detected MDV strain was named Kc-c1.

### 2.2. Polymerase Chain Reaction (PCR) and DNA Sequencing

DNA was extracted from these samples using SepaGene (Sankojunyaku, Tokyo, Japan) according to the manufacturer’s protocol. The *meq* gene was amplified using the primers MS-10 and M-AS (Table 1). The pCI-neo vectors (Promega, Madison, WI, USA) containing *meq* genes from the RB-1B and Kgw-c2 strains were used as positive controls for *meq* and S-*meq*, respectively. The PCR products were purified using Fast Gene (NIPPON Genetics, Tokyo, Japan) and submitted for sequencing analysis (Eurofins Genomics, Tokyo, Japan).

### 2.3. Construction of Expression Plasmids

The open reading frame (ORF) of the *meq* gene was amplified from the DNA samples described above using the primers EcoRI Meq and NotI Meq (Table 1), and the amplified fragments were inserted into the pCI-neo vector (Promega). The resulting expression plasmid was named pCI-VS-Meq (wt). pCI-RB-1B Meq and pCI-S-Meq (wt) carrying RB-1B *meq* and Kgw-c2 S-*meq* were used as the expression plasmids for Meq and S-Meq, respectively [22]. Amino acid substitutions were introduced into the pCI-S-Meq (wt) and pCI-VS-Meq (wt) by site-directed mutagenesis, as previously described [27], to match the amino acid sequences of S-Meq and VS-Meq with those of RB-1B Meq, except for the deletion in the proline-rich region. A glutamate-to-lysine substitution at position 77 (E77K) and a tyrosine-to-aspartate substitution at position 80 (Y80D) were introduced in VS-Meq, termed VS-Meq (RB-1B). In addition, a cysteine-to-arginine substitution at position 114 (C114R), an alanine-to-valine substitution at position 115 (A115V), and an alanine-to-proline substitution at position 176 (A176P) were introduced into S-Meq, termed S-Meq (RB-1B). The primers used for the mutagenesis are listed in Table 1. The c-Jun expression plasmid was also used for the formation of a heterodimer with Meq isoforms in reporter assays [28]. The promoter regions of pp38, which is a viral antigen expressed in cytolytic infection, Meq, and Bcl-2, which is a host molecule involved in anti-apoptotic effects, were cloned into the pGL-3 basic vector (Promega), termed pGL3b-pp38, -Meq, and -Bcl2, respectively [28], and used as reporter plasmids expressing firefly luciferase. The control reporter plasmid pRL-TK (Promega) expressing *Renilla* luciferase was used to standardize the transfection efficiency.

### 2.4. Cell Lines and Transfection

DF-1 cells (chicken fibroblast cell line), which were obtained from ATCC, were cultured in 24-well plates at 1.5 × 10^5^ cells per plate with 0.5 mL of Dulbecco’s modified Eagle’s medium (FUJIFILM Wako Pure Chemical Corp., Osaka, Japan) containing 10% fetal bovine serum (MP Biochemicals, Solon, OH, USA) and incubated at 39 °C under 5% CO_2_ for 24 h. For transfection, 300 ng/well of expression plasmids of each Meq isoform and c-Jun, 500 ng/well of each reporter plasmid, and 10 ng/well of pRL-TK were transfected using Lipofectamine 2000 (Thermo Fisher Scientific, Waltham, MA, USA) according to the manufacturer’s instructions.

### 2.5. Dual-Luciferase Reporter Assay

DF-1 cells were lysed, and luciferase activity was measured using a Dual-Luciferase Reporter Assay System (Promega) and Luminescence-JNR AB-2100 (Atto Corp., Tokyo, Japan) at 24 h post transfection. Firefly luciferase activity was standardized to *Renilla* luciferase activity, and the ratio of the luciferase activity was divided by the luciferase activity in the wells transfected with the pCI-neo vector for normalization.

### 2.6. Statistics

All values are expressed as the mean ± standard deviation. Tukey–Kramer tests were performed using R version 4.3.1 (https://www.r-project.org/, accessed on 5 October 2023). Test results with *p* < 0.05 were considered statistically significant.

## 3. Results

### 3.1. Comparison of Amino Acid Sequences of Meq

**Figure 1:**
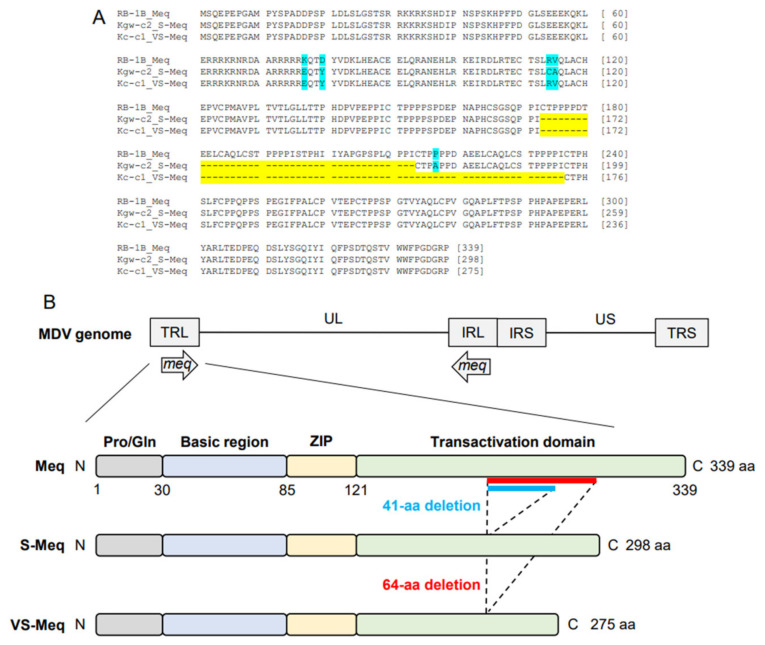
Amino acid sequences of VS-Meq and the position of the deletion. (**A**) Multiple alignment of Meq, S-Meq, and VS-Meq. Amino acid sequences of the Meq proteins from RB-1B, Kgw-c2, and Kc-c1 were compared. Deletions and polymorphisms are emphasized by a yellow and blue background, respectively. (**B**) Structure of the MDV genome and Meq protein. Meq is composed of a proline/glutamine (Pro/Gln) region, a basic region (BR), a leucine zipper (ZIP) at the N-terminus, and a transactivation domain containing proline-rich regions at the C-terminus. The proline-rich regions include PPPP motifs, and their number was different in each Meq isoform.

The length of the ORF of Kc-c1 VS-*meq*, the newly isolated sequence, was 828 base pairs (bp), which was shorter than that of the other *meq* isoforms (*meq*; 1020 bp, S-*meq*, 897 bp) (Table 2 and Table 3, Figure 1A). A 64-aa deletion was observed in the transactivation domain of Kc-c1 VS-Meq compared to Meq. This deletion was similar to that observed in a previously reported Japanese isolate (Sit-c1); however, the size of the deletion was different from that observed in a 99-35 Iranian strain (74-aa deletion) [26], MSB1 and MTB1 cells (92-aa deletion), and T cell lines derived from chickens with MD [29]. The number of PPPP/PXPP sequences in VS-Meq (99-35, and Kc-c1) was lower than that in Meq, and one PPPP sequence was present in the proline-rich regions of VS-Meq (Table 2, Figure 1B). In contrast, Meq and S-Meq contained five and three PPPP/PXPP sequences, respectively. A comparison of the amino acid sequence of Kc-c1 VS-Meq with that of Meq in RB-1B (a vv-MDV strain) detected two differences at positions 77 and 80 in BR. Glutamate at position 77 and tyrosine at position 80 are frequently observed among MDV strains circulating in the Eurasian region, including Japan [30,31,32].

### 3.2. Transrepression by VS-Meq on the pp38 Promoter

To investigate the effect of the deletion of the proline-rich region on transcriptional regulation by Meq, we examined the effects on the transactivation and transrepression of three different promoters of pp38, a viral antigen expressed in cytolytic infection, Meq, and Bcl-2, an anti-apoptotic molecule. The heterodimer with c-Jun enhances the transactivation activity of the Meq and Bcl-2 promoters [14], whereas the homodimer suppresses the activity of the pp38 promoter [10].

**Figure 2:**
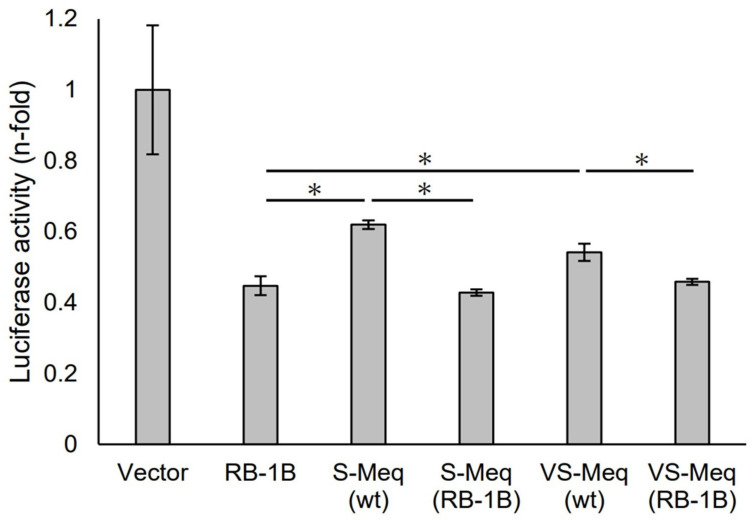
Transrepression by the deletion observed in VS-Meq on the pp38 promoter. The transrepression effects of VS-Meq (wt) and S-Meq (wt) on the pp38 promoter were compared with that of RB-1B Meq. In addition, the effects of S-Meq (RB-1B) and VS-Meq (RB-1B) were evaluated to assess the effects of the deletion. Each expression plasmid, reporter plasmid of pp38 promoter, and internal control plasmid pRL-TK were co-transfected. The firefly luciferase activities were measured and standardized using Renilla luciferase activities. Luciferase activity is expressed relative to the mean of the activities in the presence of pCI-neo vector. Two independent experiments were conducted in triplicate. Error bars indicate standard deviations. Statistical analysis was performed using the Tukey–Kramer test. Asterisks indicate significant differences (*p* < 0.05 was).

We initially evaluated the transrepression of VS-Meq on the pp38 promoter. S-Meq (wt) and VS-Meq (wt) exhibited a lower transrepression than RB-1B Meq, whereas there was no difference in the transrepression effects between S-Meq (wt) and VS-Meq (wt) (Figure 2). In addition, we investigated the effects of the deletion in the proline-rich region by reporter assays using expression plasmids that included amino acid substitutions to match the sequences of RB-1B Meq, except for the deletion, S-Meq (RB-1B), and VS-Meq (RB-1B). No differences were observed in the transrepression of RB-1B Meq, S-Meq (RB-1B), and VS-Meq (RB-1B); however, S-Meq (RB-1B) and VS-Meq (RB-1B) showed a higher transrepression of the pp38 promoter than S-Meq (wt) and VS-Meq (wt) (Figure 2). These data suggest that the deletion was not involved in the transrepression of the pp38 promoter, whereas the differences in some amino acid residues in BR seemed to affect the transrepression of Meq isoforms.

### 3.3. Transactivation by VS-Meq on the Meq and Bcl-2 Promoters

**Figure 3:**
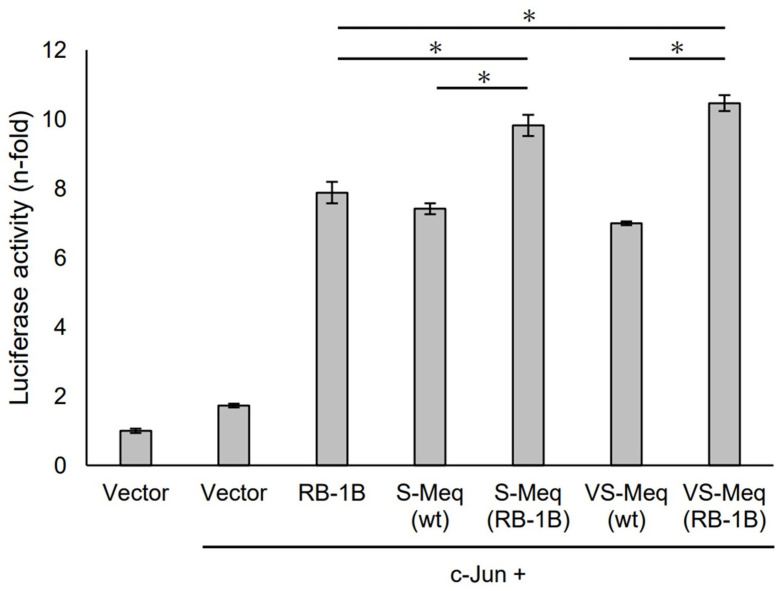
Transactivation activities by the deletion observed in VS-Meq on the Meq promoter. The effects of VS-Meq and the deletion observed in VS-Meq on the transactivation activities on the Meq promoter were compared with RB-1B Meq. S-Meq (RB-1B) and VS-Meq (RB-1B) were constructed to evaluate the effects of deletions. Expression plasmids for each Meq isoform, c-Jun expression plasmid, reporter plasmid of Meq promoter, and internal control plasmid pRL-TK were co-transfected. The firefly luciferase activities were measured and standardized using Renilla luciferase activities. Luciferase activity is expressed relative to the mean of the activities in the presence of pCI-neo vector. Two independent experiments were conducted in triplicate. Error bars indicate standard deviations. The statistical analysis was performed by the Tukey–Kramer test. Asterisks indicate significant differences (*p* < 0.05).

**Figure 4:**
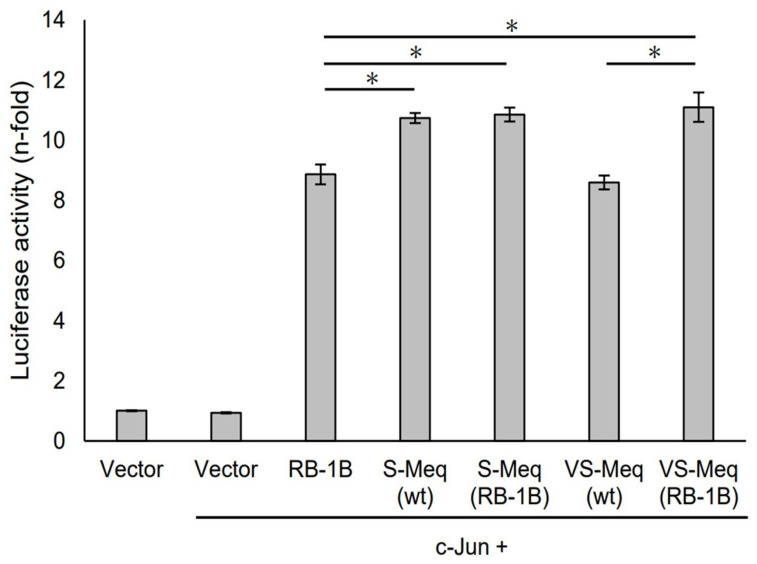
Transactivation activities by the deletion observed in VS-Meq on the Bcl-2 promoter. The effects of VS-Meq (wt) were compared with RB-1B Meq. S-Meq (RB-1B) and VS-Meq (RB-1B) were constructed to evaluate the effects of deletions. Expression plasmids for each Meq isoform, c-Jun expression plasmid, reporter plasmid of Bcl-2 promoter, and internal control plasmid pRL-TK were co-transfected. The firefly luciferase activities were measured and standardized using Renilla luciferase activities. The luciferase activity ratio is expressed relative to the mean of the activities in the presence of pCI-neo vector. Two independent experiments were conducted in triplicate. Error bars indicate standard deviations. Statistical analysis was performed by the Tukey–Kramer test. Asterisks indicate significant differences (*p* < 0.05).

S-Meq (wt) and VS-Meq (wt) did not alter the transactivation activity of the Meq promoter compared to that of RB-1B Meq (Figure 3). However, S-Meq (RB-1B) and VS-Meq (RB-1B) induced higher transactivation activities on the Meq promoter than S-Meq (wt) and VS-Meq (wt), respectively, and there were no significant differences in the transactivation activities of VS-Meq (RB-1B) and S-Meq (RB-1B) (Figure 3). Thus, the 41-aa and 64-aa deletions enhanced the transactivation activities of the Meq promoter; however, there was no difference in the level of activation between them. However, the differences in amino acid sequences between RB-1B Meq and VS-Meq (wt) /S-Meq (wt) seemed to decrease the transactivation activity. In addition, we evaluated the transactivation of the Bcl-2 promoter. VS-Meq (wt) showed no difference in the transactivation activity of the Bcl-2 promoter compared with RB-1B Meq. However, VS-Meq (RB-1B) showed a higher transactivation activity of the Bcl-2 promoter than RB-1B Meq (Figure 4). This suggested that the deletion observed in VS-Meq enhanced the transactivation activity of the Bcl-2 promoter. In contrast, S-Meq (wt) and S-Meq (RB-1B) enhanced the transactivation activity of the Bcl-2 promoter compared to RB-1B Meq. Taken together, the 41-aa deletion and 64-aa deletions have the potential to enhance the transactivation of the Meq and Bcl-2 promoters. However, the polymorphisms observed in the amino acid sequence of S-Meq (wt) appeared to be involved in the increased transactivation of the Bcl-2 promoter.

## 4. Discussion

Here, we report a case of MD in Japan caused by an MDV strain with VS-*meq*. Kc-c1 VS-Meq contained a 64-aa deletion in the transactivation domain of Meq, resulting in a decreased number of PPPP sequences. An MDV strain encoding VS-Meq has been previously detected in unvaccinated backyard chickens in Iran [26]. Kc-c1 VS-Meq was detected in the MDVs of unvaccinated chickens. However, there are no reports of MD cases caused by MDV strains with VS-Meq in vaccinated chickens. In addition, we recently reported that a 41-aa deletion in the transactivation domain of Meq reduces the virulence of MDV [17]. Therefore, the virulence of MDV encoding VS-Meq may be low. Experimental infection with recombinant MDVs (rMDVs) is required to clarify the effects of the deletion reported in this study on MDV virulence.

Kc-c1 VS-Meq (wt) showed a lower transrepression of the pp38 promoter than RB-1B Meq. Pp38 is an antigen expressed in the lytic phase of MDV infection, and Meq contributes to the establishment of MDV latency by downregulating pp38 expression [10]. Therefore, a low transrepression of pp38 promoter activity may prevent the establishment of MDV latency. In addition, Kgw-c2 S-Meq (wt) showed a lower transrepression than RB-1B Meq; therefore, a low transrepression by VS-Meq (wt) and S-Meq (wt) on the pp38 promoter may lead to decreased virulence of MDV by reducing the opportunities for target cells to be transformed during MDV latency. However, a 64-aa deletion of Kc-c1 VS-Meq and a 41-aa deletion of Kgw-c2 S-Meq did not affect the transrepression of the pp38 promoter in VS-Meq (RB-1B) or S-Meq (RB-1B). Thus, the transrepression of VS-Meq (wt) and S-Meq (wt) on the pp38 promoter seemed to decrease owing to the effect of polymorphisms in other regions of Meq but not by deletion in the proline-rich region.

The evaluation of the effects of a 64-aa deletion of Kc-c1 VS-Meq and a 41-aa deletion of Kgw-c2 on transactivation activities showed that both Meq isoforms potentially enhance the transactivation activities of Meq and Bcl-2 promoters; however, there was no difference in the level of the transactivation activities depending on the size of the deletions. Meq regulates various host genes associated with tumorigenesis, including *bcl-2* [11], which exerts anti-apoptotic effects [33]. Meq is considered to promote tumorigenesis by increasing Bcl-2 expression and enhancing anti-apoptotic effects [11]. Therefore, enhanced transactivation of the Meq and Bcl-2 promoters appears to increase MDV virulence. However, we have previously reported that rMDV encoding a 41-aa deletion in Meq exhibited a lower virulence than that of rMDV with Meq, although it caused a higher transactivation activity on the Meq promoter than Meq [17,22]. Therefore, a 64-aa deletion in Meq may result in low virulence, similar to the 41-aa deletion in Meq. It is unclear how these deletions in Meq increase the transactivation activities of the Meq and Bcl-2 promoters; however, deletion in the proline-rich regions of Meq may affect its interaction with other proteins that regulate transcription, such as transcriptional factors and activators. Moreover, Meq exhibits multiple functions through its interactions with other proteins such as p53, retinoblastoma protein, heat shock protein 70, cyclin-dependent kinase 2, C-terminal-binding protein, histone deacetylases 1 and 2, STING, and interferon regulatory factor 7 (IRF7) [12,34,35,36,37,38,39]. In particular, Meq impairs IFN-β production by interacting with STING and IRF7 via the transactivation domain where the deletions were found. Therefore, deletions in the transactivation domain potentially affect innate immune responses and subsequently alter the virulence of rMDV with Meq, including a 41-aa deletion. In addition, Meq can suppress apoptosis by inhibiting p53 transcriptional activity via interaction with the N-terminus and bZIP region [34]. Although Meq and S-Meq containing a 41-aa deletion could inhibit p53 transcriptional activity, Meq containing a 92-aa deletion in the transactivation domain showed no inhibitory effect on p53 transcriptional activity [34]. Thus, the 64-aa deletion may affect MDV virulence by inhibiting p53-induced apoptosis. Further investigation is needed to determine whether a 64-aa deletion in the proline-rich region of Meq alters MDV virulence and to clarify the underlying molecular mechanisms.

## Figures and Tables

**Table 1:** Primers used for the sequence analysis and the construction of expression plasmids.

Primer Name	Purpose	Sequence (5′-3′)
MS-10	Sequencing	ATGTCTCAGGAGCCAGAGCCGGGCGCT
M-AS	Sequencing	GGGGCATAGACGATGTGCTGCTGAG
EcoRI Meq	Cloning into pCI-neo vector	CCCGAATTCATGTCTCAGGAGCCAGAGCCGGGCGCT
Not I Meq	Cloning into pCI-neo vector	ATAAGAAATGGGGCCGCGGCGGGGCATAGACGATGTGCTGCTGAG
E77K Fw	Mutagenesis	AGGAAGCAGACGTACTATGTAGACA
E77K Rv	Mutagenesis	TGTCTACATAGTACGTCTGCTTCCTT
Y80D Fw	Mutagenesis	AGAAGACGCAGGGAGCAGACGGACT
Y80D Rv	Mutagenesis	AGTCCGTCTGCTCCCTGCGTCTTC
E77K Y80D Fw	Mutagenesis	AGAAGACGCAGGAAGCAGACGGACT
E77K Y80D Rv	Mutagenesis	AGTCCGTCTGCTTCCTGCGTCTTCT
C114R A115V Fw	Mutagenesis	GAGTGCACGTCCCTGCGTGTACAGTTGGCTTGT
C114R A115V Rv	Mutagenesis	TGACAAGCCAACTGTACACGCAGGGACGTGCACTC
A176P Fw	Mutagenesis	ATCTGTACCCCCGCTCCTCCCGATA
A176P Rv	Mutagenesis	TATCGGGAGGAGCGGGGGTACAGAT

**Table 2:** Information of the S-Meq and VS-Meq.

Strain	Isoform	Country	Accession No.	Length			Number of PPPP
				ORF (bp)	Amino Acid (aa)	Deletion (aa) ^d^	
RB-1B	Meq	USA	EF523390	1020	339	0	5
CVI988	S-Meq	Netherlands	AY243338	897	298	41	3
855/17	S-Meq	Italy	MK139678	897	298	41	2
MDV/2/SA ^a^	S-Meq	Saudi Arabia	LC385871	897	298	41	3
Iraq3A	S-Meq	Iraq	KC243262	897	298	41	2
Iraq10A	S-Meq	Iraq	KC243264	897	298	41	2
Iraq6F	S-Meq	Iraq	KC243263	897	298	41	2
99-26	S-Meq	Iran	MZ962187	897	298	41	2
99-35	VS-Meq	Iran	MW990216	798	265	74	1
VS-Meq ^b^	VS-Meq	Japan	AB087744	744	247	92	1
Nr-c1 ^c^	Meq	Japan	LC385871	1020	339	0	3
Kgw-c2	S-Meq	Japan	CL385874	897	298	41	2
Sit-c1	VS-Meq	Japan	BBE28998	828	275	64	1
Kc-c1	VS-Meq	Japan	LC790435	828	275	64	1

^a^ The MDV/2/SA S-Meq sequence is partially complete. ^b^ The sequences were detected in MSB1 and MTB1 cells. ^c^ The sequence is most frequently observed in recent Japanese isolates. ^d^ All deletions were detected in the proline-rich regions.

**Table 3:** Comparison of amino acid sequences of Meq.

	Basic Region						Leucine Zipper						Transactivation Domain							
Strain	36	70	71	77	78	80	88	100	110	114	115	119	141	168	176 *	217 *	218 *	221 *	281 *	326 *
RB-1B	S	A	A	K	Q	D	A	R	C	R	V	C	H	S	P	P	P	A	G	T
CVI988	S	A	A	E	Q	D	A	R	C	R	V	C	H	S	-	P	P	A	G	I
855/17	S	A	A	E	Q	Y	A	R	S	R	V	C	H	S	-	P	S	T	G	T
MDV/2/SA	S	A	A	E	Q	Y	A	R	S	R	V	C	P	S	-	P	P	A	G	T
Iraq3A	S	A	A	E	Q	Y	A	R	C	R	V	R	H	P	-	A	P	A	G	T
Iraq10A	S	A	S	E	Q	D	A	R	C	R	V	C	H	P	-	A	P	A	G	T
Iraq6F	S	A	S	E	Q	D	A	R	C	R	V	C	H	S	-	A	P	A	G	T
99-26	T	A	A	K	H	D	A	R	C	R	V	R	H	S	-	A	P	A	D	T
99-35	S	A	A	E	Q	Y	T	R	C	R	A	C	H	S	-	-	-	-	G	T
VS-Meq	S	S	A	K	Q	D	A	H	C	R	V	C	H	S	-	-	-	-	G	T
Nr-c1	S	A	A	E	Q	Y	A	R	C	R	V	C	H	S	S	A	P	A	G	T
Kgw-c2	S	A	A	E	Q	Y	A	R	C	C	A	C	H	S	-	A	P	A	G	T
Sit-c1	S	A	A	E	Q	Y	A	R	C	R	V	C	H	S	-	-	-	-	G	T
Kc-c1	S	A	A	E	Q	Y	A	R	C	R	V	C	H	S	-	-	-	-	G	T

* Amino acid positions are shown in Meq.

## Data Availability

The data presented in this study are available in the article.
